# Supplementary material for: Factors associated with Anganwadi Workers’ service delivery of Integrated Child Development Services (ICDS) in rural India: A cross-sectional analysis of household and community health worker surveys
Source: PLoS One. 2025 Jul 18;20(7):e0326971. doi: 10.1371/journal.pone.0326971 (PMC12273970; doi:10.1371/journal.pone.0326971)
Supplement: S1 Table — (DOCX) [file pone.0326971.s001.docx]

**Table S1: Detailed construction of outcome indicators and definitions**

| **Variable** | | **n** | **Type** | **Definition** |
| --- | --- | --- | --- | --- |
| ***Product-Oriented Service Delivery*** | | | | |
| Growth monitoring | Weighing of children 0-12 months | 6635 | Binary | 1 if the mother reported that the AWW weighed her child at least once in the last 3 months, 0 otherwise |
| Take home rations | Received THR for woman/child one or more times a month from AWC | 9033 | Binary | 1 if the pregnant woman / mother reported that she receives THR one or more times a month from the AWC, and 0 otherwise. |
| Hot cooked meals | Received hot cooked meals for herself at the AWC | 2398 | Binary | 1 if the pregnant woman reported that she receives hot cooked meals at the center, 0 otherwise. |
| ***Information-Oriented Service Delivery*** | | | | |
| Adequate home visits | Quantity of home visits received from AWWs | 9033 | Binary | ICDS has detailed guidelines specifying the number of home visits a beneficiary should receive at different life stages. For example, a mother must receive at least one home visit per month when the child is between 6-8 months of age. The indicator is coded 1 if the mother/pregnant woman received adequate number of visits in the last three months, and 0 otherwise. An additional assumption was added that every woman should receive at least one home visit in each 3-month period. |
| Counseling on nutrition | Received counseling on ICYF/nutrition from AWWs | 9033 | Binary | 1 if the pregnant woman/ mother reported receiving counseling on infant feeding (on exclusive breastfeeding, right time to start complementary feeding, dietary diversity) |
