# Supplementary material for: Factors associated with Anganwadi Workers’ service delivery of Integrated Child Development Services (ICDS) in rural India: A cross-sectional analysis of household and community health worker surveys
Source: PLoS One. 2025 Jul 18;20(7):e0326971. doi: 10.1371/journal.pone.0326971 (PMC12273970; doi:10.1371/journal.pone.0326971)
Supplement: S2 Table — (DOCX) [file pone.0326971.s002.docx]

**Table S2: Supplementary Table showing frequencies and percentages of services received by beneficiaries based on AWW’s means, motives, and opportunity variables**

|  | **Population** | **Growth monitoring** | **Take home rations** | **Hot cooked meals** | **Adequate number of home visits** | **Counseling on IYCF** |
| --- | --- | --- | --- | --- | --- | --- |
|  |  | **Children 0-12 months**  **% (n)** | **Pregnant women and**  **mothers of children 0-12m**  **% (n)** | **Pregnant women only**  **% (n)** | **Pregnant women and Mothers of children 0-12 months**  **% (n)** | |
|  | Total sample (N) | 6635 | 9033 | 2398 | 9033 | 9033 |
|  | Beneficiaries received services in columns mentioned (n) | 47.5% (3148) | 52.2% (4716) | 20.3% (488) | 37.2% (3363) | 45.0% (4070) |
| **Means** | | | | | | |
| **Education** | 10 or more years of education | 45.8% (2508) | 49.5% (3723) | 19.7% (403) | 36.1% (2710) | 43.4% (3262) |
|  | Less than 10 years of education | 55.1% (640) | 65.5% (993) | 24.1% (85) | 43.1% (653) | 53.3% (808) |
| **Experience** | AWW with more than 14 years of experience | 47.6% (1751) | 52.7% (2624) | 20.6% (270) | 37.3% (1857) | 46.1% (2298) |
|  | AWW with less than 14 years of experience | 47.0% (1397) | 51.6% (2092) | 20.1% (218) | 37.1% (1506) | 43.7% (1772) |
| **Knowledge** | AWW with mean knowledge score more than 30 | 48.9% (1834) | 51.9% (2662) | 20.9% (287) | 37.9% (1941) | 46.1% (2360) |
|  | AWW with mean knowledge score less than 30 | 45.6% (1314) | 52.5% (2054) | 19.5% (201) | 36.4% (1422) | 43.7% (1710) |
| **Skills** | AWW with mean skills score more than 13 | 50.4% (1680) | 54.9% (2480) | 22.1% (262) | 39.3% (1776) | 48.6% (2200) |
|  | AWW with mean skills score less than 13 | 44.4% (1468) | 49.5% (2236) | 18.6% (226) | 35.1% (1587) | 41.4% (1870) |
| **Motives** | | | | | | |
| **Motivation** | AWW who were motivated | 48.2% (2952) | 53.3% (4438) | 21.1% (463) | 37.7% (3144) | 46.2% (3847) |
|  | AWW who were not motivated | 38.7% (196) | 39.0% (278) | 12.2% (25) | 30.7% (219) | 31.3% (223) |
| **Timely salary** | AWW who received timely salary | 60.1% (962) | 68.1% (1449) | 25.1% (132) | 45.5% (969) | 56.8% (1209) |
|  | AWW who did not receive timely salary | 43.4% (2186) | 47.3% (3267) | 19.0% (356) | 34.6% (2394) | 41.4% (2861) |
| **Supervision** | AWW who received supervision | 45.8% (1717) | 51.1% (2617) | 21.9% (302) | 37.4% (1920) | 44.7% (2295) |
|  | AWW who did not receive supervision | 49.5 (1431) | 53.7% (2099) | 18.2% (186) | 36.9% (1443) | 45.4% (1775) |
| **Opportunities** | | | | | | |
| **Caste** | SC/ST AWW | 47.9% (2358) | 52.1% (3479) | 20.9% (370) | 37.6% (2515) | 44.3% (2962) |
|  | Non SC/ST AWW | 45.9% (790) | 52.5% (1237) | 18.6% (118) | 36.0% (848) | 47.1% (1108) |
| **Infrastructure** | AWC has salter scale for weighing | 49.2% (2893) |  |  |  |  |
|  | AWC does not have salter scale for weighing | 33.9% (255) |  |  |  |  |
| **Storage** | AWC has THR storage |  | 56.4% (3149) | 21.7% (312) |  |  |
|  | AWC has no THR storage |  | 45.4% (1567) | 18.2% (176) |  |  |
| **Supplies** | AWW has THR supply issues |  | 39.3% (902) | 19.3% (121) |  |  |
|  | AWW has no THR supply issues |  | 56.6% (3814) | 20.7% (367) |  |  |
| **Training** | AWW is adequately trained | 57.8% (1966) | 63.7% (2909) | 27.5% (321) | 42.4% (1936) | 54.0% (2466) |
|  | AWW is not adequately trained | 36.5% (1182) | 40.4% (1807) | 13.5% (167) | 31.9% (1427) | 35.8% (1604) |
| **Workload** | AWW has higher workload | 31.9% (972) | 36.9% (1577) | 14.5% (179) | 29.5% (1264) | 34.7% (1481) |
|  | AWW has lighter workload | 60.5% (2176) | 65.9% (3139) | 26.4% (309) | 44.1% (2099) | 54.3% (2589) |
