# Supplementary material for: Factors associated with Anganwadi Workers’ service delivery of Integrated Child Development Services (ICDS) in rural India: A cross-sectional analysis of household and community health worker surveys
Source: PLoS One. 2025 Jul 18;20(7):e0326971. doi: 10.1371/journal.pone.0326971 (PMC12273970; doi:10.1371/journal.pone.0326971)
Supplement: Attachment — Submitted filename: Response to Reviewers.docx [file pone.0326971.s004.docx]

Dear Reviewer and Editor:

We appreciate the thorough and constructive feedback provided by the reviewer and the editor. We are particularly encouraged by reviewer’s positive assessment that “*The authors have chosen a very relevant topic and have meticulously conducted extensive background work on the same*.” Further, the editor too noted: “*The article covers a very important topic of immediate relevance”.* We have carefully considered all comments and suggestions and have made appropriate revisions to strengthen the manuscript. Our responses are in red below. Page numbers where we made edits are highlighted in yellow in the track change version to easily find the edits.

Reviewer #1: The authors have chosen a very relevant topic and have meticulously conducted extensive background work on the same. They have identified various domains of determinants and outcomes for addressing this research question. This is a descriptive study with an analytical component to look for factors associated with the service delivery of Integrated Child Development Services (ICDS). Secondary data analysis has been done. There has been no data collection specifically to answer this research question. The paper has been written very well.
A few queries
1. The Caste variable – it has been speculated in the article that the reason AWWs from SC/ST backgrounds are unable to perform their responsibilities concerning growth monitoring and home visits is because of caste dynamics. However, it has also been mentioned in the paper that the model was adjusted for Caste status. It is a very sensitive topic and needs to be treated carefully.

Response to reviewer: Thank you for this insight. Yes, you’re right that we controlled for household caste and still found that AWW caste was associated with service delivery outcomes, so we have now revised our discussion of this finding to more carefully present the possible interpretations.

First, we acknowledge the fact that we controlled for household caste. Our findings indicate that AWWs from Scheduled Caste/Scheduled Tribe backgrounds had lower odds of providing growth monitoring services and conducting home visits, even after controlling for household caste. We further added:

“This observed association may reflect complex social dynamics that quantitative analysis alone cannot fully explain. While previous qualitative research from Bihar (John et al., 2020) suggests that caste-based social hierarchies can affect service delivery, other unmeasured factors correlated with AWW caste may also contribute to these differences. Future research using mixed methods would be valuable to better understand the specific mechanisms through which AWW caste relates to service delivery, particularly examining how institutional, community, and interpersonal factors interact to create barriers or facilitators for AWWs from different caste backgrounds.”

Please refer to Page 14 of 19 for revised discussion paragraph on caste dynamics.

2. The paper has identified that ‘training’ is an essential determinant of the service delivery of ICDS. However, the methods section does not elaborate on what this training entails. Is it ever trained/never trained? Or Trained in the last 1-year vs more than a year ago? Also the contents of the training, taught by whom etc. This is essential as it has become the single most influential factor across domains.

Response to reviewer: Thank you for highlighting this important point. In the revised manuscript, we have included this detailed explanation in the methods section to provide readers with a clearer understanding of how this key variable was operationalized, especially given its significant association with service delivery outcomes across multiple domains. Page 6 of 18 clearly defines how training variable was coded.

To clarify, our training variable was defined as an indicator variable coded as 1 if the AWW reported receiving training on all 23 modules within the 12 months prior to the survey, and 0 if they received training on some but not all modules. This binary categorization ("Trained in all" vs. "Trained in some") allowed us to assess the impact of comprehensive training coverage. The 23 training modules delivered by supervisors covered a broad range of AWW responsibilities including:

1. **Nutritional services**: Growth monitoring, complementary feeding, management of malnutrition (SAM), feeding during illness, maternal nutrition during pregnancy and lactation
2. **Maternal and child health**: Antenatal care, postnatal care, breastfeeding, immunization, family planning, identification of sick newborns
3. **Community health activities**: Organizing Village Health and Nutrition Days (VHNDs), home visit scheduling, referrals to health centers
4. **Other topics**: Hygiene, sanitation, adolescent health

Our approach focused on comprehensiveness of training rather than recency, as all training modules were delivered within the previous 12 months.

3. Would it be reasonable to say that the study concludes that for an AWW to function well, they must be educated for more than 10 years? Therefore, can that be a criterion to consider when choosing AWWs? It is a thought for the authors to ponder.

Response to reviewer: Thank you for this suggestion. Please refer to Page 13 of 18 under Motives paragraph.

Our findings do indicate that AWWs with 10 or more years of education had 30% higher odds of providing growth monitoring services compared to those with less education. This aligns with some previous literature suggesting education may enhance CHW performance. However, based on these results alone, it would not be possible for us to recommend education as a strict criterion for reasons such as:

1) Education was significantly associated only with growth monitoring services, not with other key services like supplementary food provision, home visits, or counseling. This suggests the relationship between AWW education and performance are service-specific rather than universal.
2) Further, we feel that raising the minimum educational requirements could inadvertently exclude capable candidates from communities with limited educational access for women, potentially reducing community representation among AWWs. Such requirements may pose challenges in recruitment and retention of AWWs.
3) Finally, our findings indicate that opportunity-related factors (training, supervision, adequate supplies) were more consistently associated with AWW performance across multiple services than educational background. These modifiable program factors may offer more actionable paths to improving service delivery.

Addressed this by adding a point on the importance of education but also cautioning the readers about relying solely on education as criteria for performance.

4. As mentioned in the paper, information bias is a significant worry in this particular study design. The paper has also been completed 5 years after the original study concluded. This may add to the bias. The question on ‘motivation’ is bound to have a substantial social desirability bias. In addition, there is a possibility of Hawthorne bias as this was a part of another study on the usage of a mobile app.

Response to reviewer:

Thank you for highlighting these important methodological considerations. We acknowledge several potential sources of bias in our study:

1. **Information bias**: We recognize the limitations of self-reported data from both AWWs and beneficiaries. To minimize recall bias, we focused on recent service delivery experiences and incorporated data from multiple stakeholders (mothers, pregnant women, and AWWs).
2. **Social desirability bias**: We acknowledge that measures such as AWW motivation likely reflect some degree of social desirability bias. We attempted to mitigate this by using trained female enumerators and privacy during interviews, but recognize this remains a limitation, particularly for subjective measures.
3. **Timing of analysis**: While the data collection was completed in 2018-19 and the analysis conducted later, this temporal gap does not affect the internal validity of the associations we report. The data itself represents conditions at the time of collection, and the relationships between AWW characteristics and service delivery outcomes remain valid regardless of when the analysis was performed.
4. **Hawthorne effect**: As this study was embedded within a larger evaluation of an mHealth intervention, but the mHealth intervention was with the AWWs. However, it is possible AWWs and even to an extent, participants may have altered their behavior due to awareness of being observed. We attempted to control for this by including district fixed effects in our models, as the intervention was assigned at the district level. However, we cannot completely rule out potential reactivity effects.

Despite these limitations, the consistency of our findings with qualitative research from similar contexts and the robust associations observed across multiple outcome measures suggest that our key findings regarding training, supervision, and resource availability as determinants of AWW performance remain valid and informative for program implementation. We have addressed this in the limitations section of the paper (Page 14 of 19) in the track changes version.

5. It would have been more illuminating to have the actual frequencies and percentages in addition to the Odds Ratios.

We have now added a supplementary table (Supplementary Table S2) showing the percentage and number of beneficiaries receiving each service stratified by key AWW characteristics that align with our Means, Motives, and Opportunity framework. This descriptive analysis complements our regression results.

**Additional Editor Comments:**

Thank you for your thoughtful comments on our article. We have carefully addressed each point to strengthen the paper:

1. Mode of recruitment of AWW decide on their capability, and also play a huge influence on their motivation. Can a para be added on government policy and practice on how AWW are recruited, whether Recruitment Rules are framed and followed.

We have added a detailed paragraph on AWW recruitment policies in the introduction (**Page 2 of 18, Paragraph 2**): “AWWs are selected from local villages by State-appointed committees, with minimum qualification requirements of Matriculation (10th standard) and age limits of 18-35 years. As honorary workers rather than formal employees, AWWs receive modest honoraria (₹4,500 monthly plus ₹500 performance-linked incentive as of 2021), which may be supplemented by state governments. This recruitment approach directly influences both AWW capabilities and motivation - local recruitment enhances community embeddedness but potentially limits the educational qualifications of candidates, while the honorary worker status and compensation structure may affect motivation and retention, particularly given the substantial responsibilities assigned to AWWs (Press Information Bureau, 2021).”

2. Compare data on services with that from NFHS-5.

We have incorporated a comprehensive comparison with NFHS-5 data in our discussion section (Page 12 of 18, Paragraph 2). This comparison contextualizes our findings within national survey data: “Our findings on ICDS service coverage show some alignment with patterns observed in NFHS-5 data, though with notable variations. In Madhya Pradesh and Bihar, NFHS-5 reported that 75.9% and 41.3% of children under 6 years, respectively, received supplementary food through ICDS, averaging to 58.6% across both states - somewhat higher than our observed THR receipt rates (52.2%). For pregnant women, NFHS-5 found that 43.8% in Bihar and 83.8% in MP received supplementary food during pregnancy, substantially higher than the hot cooked meals (20.4%) reported in our study. This difference likely reflects that NFHS-5 defines supplementary food to include both food cooked and served at the AWC and take-home rations. Regarding growth monitoring, NFHS-5 showed that 35.2% in Bihar and 77.8% in MP of children were weighed by AWWs, compared to 47.5% in our sample, placing our findings between the two states’ averages. NFHS-5 does not provide data on home visits or nutrition counseling, preventing comparison for these services. These patterns suggest regional variations in ICDS implementation, with our findings generally reflecting the broader service delivery landscape across these states (International Institute for Population Sciences, 2022).”

3. Caste composition of served population is relevant when evaluating performance of AWW.

Our methodology explicitly accounts for the caste composition of the served population. As noted in our methods section (Page 7 of 18, Paragraph on Covariates), we controlled for household caste in all regression models, using an indicator variable for historically marginalized scheduled caste/scheduled tribe (SC/ST) households. Additionally, our analysis of AWW caste factors take into account this control for beneficiary caste, allowing us to identify associations between AWW characteristics and service delivery independent of the population served. This methodological approach addresses the important social dynamics you highlighted.

We believe these additions have significantly strengthened the paper while maintaining its focus on the factors associated with AWW service delivery. Thank you for your valuable feedback that has enhanced the relevance and strengthened our paper.
