# Supplementary material for: Factors associated with Anganwadi Workers’ service delivery of Integrated Child Development Services (ICDS) in rural India: A cross-sectional analysis of household and community health worker surveys
Source: PLoS One. 2025 Jul 18;20(7):e0326971. doi: 10.1371/journal.pone.0326971 (PMC12273970; doi:10.1371/journal.pone.0326971)
Supplement: Attachment — Submitted filename: Response_to_Reviewers_auresp_2.docx [file pone.0326971.s005.docx]

Dear Editor:

We appreciate the thorough and constructive feedback provided by the editor. We have carefully considered all comments and suggestions and have made appropriate revisions to strengthen the manuscript. Our responses are in red below along with page numbers. Edits are highlighted in yellow in the track change version.

Additional Editor Comments:

Appreciate the constructive manner of responding to reviewers comments, and making amends in the manuscript. The article as it exists stands adds to existing knowledge on this crucial subject. There are some suggestions (listed below) for the consideration of the authors for further improvement of the piece.

1. Intro: "Each AWW caters to a catchment area of approximately 800–1000 children under the age of six and their pregnant or breastfeeding mothers." Please recheck this fact, as program manual mentions of a POPULATION of 1000 for each cluster under the AWW. This also matches with the statement in results that "AWWs typically had around 73 beneficiaries to serve in their villages"

Edited this to 1000 on Page 3 of 18.

2. Introduction: "delivering the ICDS services: growth monitoring activities of children as appropriate; delivering supplementary food including hot cooked meals and take-home rations; conducting home visits and counseling to educate pregnant and lactating women on pregnancy care, and infant and young child feeding practices". Best to cite the standard six ICDS services here, in Figure -1, and elsewhere as these are the core ICDS services that AWW are expected to deliver.,
Noted and cited.

3. Similarly, the six outcome variables of "Product-oriented performance outcomes in the study" miss the following of the six services under ICDS pre-school education, health check-ups, immunization, referral services.
To make the findings complete and generalizable, some explanation on why there services were left out is required.
We agree with you that not all the ICDS services are covered. Our study deliberately focused on specific nutrition-related services provided by Anganwadi Workers for several methodologically sound reasons:

1) Our study was focused on services critical to addressing malnutrition in the first 1000 days of life - a period widely recognized as the most crucial window for nutritional intervention. Growth monitoring, supplementary food distribution, home visits, and IYCF counseling represent the core nutrition-focused services that have the strongest evidence base for impact on child growth and development outcomes during this critical period. This was also the overlap with the CAS Application (mHealth intervention) designed for AWWs, and within the evaluation, we asked other questions that helped us examine research questions in this paper. Therefore, our analytical sample comprised 6,653 mothers of children below 12 months and 2,398 pregnant women. This population was specifically selected to examine services targeting pregnancy and early infancy, which are primarily nutrition-focused. Pre-school education services would require data from families with children aged 3-6 years, which was outside the scope of our targeted population.
2) While AWWs are involved in multiple ICDS services as outlined in the guidelines, not all services fall exclusively under their direct responsibility. For example:
   a. Immunization services are typically delivered through a collaborative effort involving ASHAs and ANMs during Village Health and Nutrition Days, with AWWs playing a supportive rather than primary role
   b. Referral services represent an outcome of other service interactions rather than a distinct service delivery action. Growth monitoring is one aspect of referral services. Based on the category of malnutrition, children are then referred by AWW, though she does not have the sole authority to ensure children end up in nutrition rehabilitation centers.
3) We were conducting the secondary data analysis from an endline evaluation of an mHealth intervention specifically designed to digitally enable AWWs in delivering nutrition services. The original data collection instruments were therefore calibrated to measure the nutrition-related services that the intervention was intended to enhance.

I have added this to the limitation on page 14 of 18 : An additional limitation is that we did not examine the full spectrum of ICDS services. We focused specifically on nutrition-related services targeting the first 1000 days of life (growth monitoring, supplementary food, home visits, and IYCF counseling), which aligned with our study population of mothers with children under 12 months and pregnant women. Pre-school education services would have required data from families with children aged 3-6 years. Services like immunization (delivered collaboratively with ASHAs and ANMs) and referral services (which represent outcomes rather than distinct service actions) were outside our scope. Our results are not generalizable to the full range of services offered by the AWWs.

4. Since the data was collected at endline after a mHealth intervention, how generalizable can it to be to the conditions across ICDS project areas in general ?

While acknowledging that the data was collected in a post-intervention context, we had the following methodological strengths that support the generalizability of our findings to the broader ICDS system.

Added this limitation on page 14 of 18: We used the full dataset of both intervention and control districts to retain the full sample and addressed this by controlling for the district fixed effects as the intervention was delivered at the district level.This statistical approach effectively controlled for any potential influences of the mHealth intervention, which was delivered at the district level. By incorporating these fixed effects, we essentially compared AWWs within the same sub-districts, thereby accounting for both intervention-related factors and sub-district level characteristics that might influence service delivery patterns or women’s health-seeking behaviors. Further, by using beneficiary-reported outcomes rather than program administrative data, our measures capture actual service receipt from the perspective of intended beneficiaries, which provides a more realistic assessment of service delivery irrespective of the intervention. This approach helps mitigate potential reporting biases that might arise from AWWs themselves in an intervention context.

5. Since around 19% of AWW were not matriculate, authors may check if this was indeed a necessary qualification.

Thank you for this insightful observation. The paper states that the minimum qualification requirement for AWWs is matriculation (10th standard), as per official ICDS guidelines. However, as correctly noted, our data shows that approximately 19% of AWWs in our sample had not completed this level of education.

This discrepancy likely reflects the practical reality of AWW recruitment in rural MP and Bihar, where policy implementation often differs from written guidelines. The ICDS program guidelines do specify matriculation as the minimum qualification, but states have historically been granted flexibility in implementation, especially in areas where finding qualified candidates willing to work for the provided honorarium has been challenging. This finding actually reinforces one of our key points about the tension between local recruitment (which enhances community embeddedness) and educational qualifications of candidates. The presence of AWWs without matriculation in our sample provides an opportunity to examine whether this official educational requirement is indeed necessary for effective service delivery. Interestingly, our analysis showed that higher education (10 or more years) was significantly associated only with growth monitoring services but not with other service outcomes. This suggests that while education may be beneficial for technically complex tasks, it may not be uniformly necessary across all AWW responsibilities, which has important implications for recruitment policies in resource-constrained settings.

I added this note in the discussion on Page 12 of 18 “This suggests that while the matriculation (completing tenth grade) requirement may be beneficial for certain technical aspects of the AWW role, it may not be uniformly necessary across all responsibilities. This finding has important implications for AWW recruitment policies, particularly in areas with limited candidate pools, and suggests that task-specific training and supportive supervision may potentially compensate for lower formal education in some aspects of service delivery.”
