# Supplementary material for: Factors associated with Anganwadi Workers’ service delivery of Integrated Child Development Services (ICDS) in rural India: A cross-sectional analysis of household and community health worker surveys
Source: PLoS One. 2025 Jul 18;20(7):e0326971. doi: 10.1371/journal.pone.0326971 (PMC12273970; doi:10.1371/journal.pone.0326971)
Supplement: Attachment — Submitted filename: Response_to_Reviewers_auresp_3.docx [file pone.0326971.s006.docx]

Dear Editor:

We appreciate the thorough and constructive feedback provided by the editor. We have carefully considered all comments and suggestions and have made appropriate revisions to strengthen the manuscript. Our responses are in red below along with page numbers. Edits are in the track change version.

Additional Editor Comments:

1. Population served by AWW:
Its 1000 people, not 1000 children, as has erroneously appeared in your latest draft.
Apologies for missing it earlier. Edited it now.

2. Hot Cooked Meals
The text and table refers to only Pregnant Women. Why is the data for eligible children (6 months to 6 years) not included ?

The text and table refer only to pregnant women because ICDS provides THR to children 6 months to 3 years. Hot cooked meals are offered to children attending the AWC (3– 6 years). Our sample comprised 6,653 mothers of children below 12 months and 2,398 pregnant women, totaling 9,051 participants. This was because our study was specifically designed to examine services around maternal nutrition services and interventions targeting pregnancy and early infancy.

How it is address in the manuscript:

I have captured the above point in two places – one as a note to clarify that hot cooked meals are only given to children 3-6years on Page 7 of 18 in track change version.

Further in the limitations section we have mentioned our focus on the first 1000 days of life and nutrition services for women around pregnancy. “Our data collection instruments were developed to capture services primarily delivered to mothers rather than direct child feeding programs at AWC. We focused only on nutrition-related services targeting the first 1000 days of life (growth monitoring, supplementary food, home visits, and IYCF counseling), which aligned with our study population of mothers with children under 12 months and pregnant women. Pre-school education services and cooked meals for children at AWC would have required data from families with children aged 3-6 years. Services like immunization (delivered collaboratively with ASHAs and ANMs) and referral services (which represent outcomes rather than distinct service actions) were outside our scope. Our results are not generalizable to the full range of services offered by the AWWs. Future studies should examine services around the full range of ICDS services.*” Please see on Page 14 of 18 in track change version*
